# Theoretical 3D electron diffraction electrostatic potential maps of proteins modeled with a multipolar pseudoatom data bank

**DOI:** 10.1107/S2059798322005836

**Published:** 2022-07-14

**Authors:** Marta Kulik, Michał Leszek Chodkiewicz, Paulina Maria Dominiak

**Affiliations:** a Biological and Chemical Research Centre, Faculty of Chemistry, University of Warsaw, Zwirki i Wigury 101, 02-089 Warsaw, Poland

**Keywords:** electron diffraction, transferable aspherical atom model, multipolar scattering factors, cryo-electron microscopy, quantum crystallography, electrostatic potential maps, UBDB, macromolecular structure, proteins

## Abstract

The availability of atomic resolution experimental maps of electrostatic potential from 3D electron diffraction (3D ED) extends the possibility of investigating the electrostatic potential beyond the determination of non-H-atom positions. However, accurate tools to calculate this potential for macromolecules, without the use of expensive quantum calculations, are lacking. The University at Buffalo Data Bank (UBDB) gathers atom types that can be used to calculate accurate electrostatic potential maps via structure-factor calculations. Here, the transferable aspherical atom model (TAAM) is applied with UBDB to investigate theoretically obtained electrostatic potential maps of lysozyme and proteinase K, and compare them with experimental maps from 3D ED. UBDB better reproduces the molecular electrostatic potential of molecules within their entire volume compared with the neutral spherical models used in the popular independent atom model (IAM). Additionally, the theoretical electron-density maps of the studied proteins are shown and compared with the electrostatic potential maps. The atomic displacement parameters (*B* factors) may affect the electrostatic potential maps in a different way than in the case of electron-density maps. The computational method presented in this study could potentially facilitate the interpretation of the less resolved regions of cryo-electron microscopy density maps and pave the way for distinguishing between different ions/water molecules in the active sites of macromolecules in high-resolution structures, which is of interest for drug-design purposes.

## Abstract 2

Accurate electrostatic potential maps and electron-density maps of proteins are calculated based on the transferable aspherical atom model using a pseudoatom data bank and are compared with the experimental data.

## 1. Introduction

The enormous advances in the field of cryo-electron microscopy (cryo-EM) have expanded the possibility of obtaining high-resolution structures (Wu & Lander, 2020 ▸; D’Imprima & Kühlbrandt, 2021 ▸). Similar progress is noticeable in 3D electron crystallography (3D ED) methods, in particular in microcrystal electron diffraction (microED), which uses crystals with submicrometre thicknesses as samples (Shi *et al.*, 2013 ▸; Nannenga *et al.*, 2014 ▸; Nannenga & Gonen, 2018 ▸). At the same time, deep understanding and theoretical modeling of the density maps derived in all these experiments lags behind.

From the physical point of view, the observed density is the finite resolution Fourier image of the true electrostatic potential of the studied sample. A map of the same electrostatic potential should be extracted from 3D ED experiments. The electrostatic potential map is shaped by the electrons scattered by both the positively charged atom nuclei and the negatively charged electron cloud. In contrast, the electron-density maps obtained in X-ray crystallography are only shaped by X-rays scattered by the negatively charged electron cloud (Marques *et al.*, 2019 ▸). It is worth noting that electrons are scattered by matter more efficiently than X-rays (Dorset, 1991 ▸), thus smaller amounts of sample and shorter exposures to radiation are needed in electron diffraction than in X-ray diffraction experiments. The scattering of electrons depends on atomic charges and scattering angles: the amplitudes are always positive for non-negatively charged atoms, but for negatively charged atoms at low scattering angles the amplitude values become negative (Marques *et al.*, 2019 ▸; Jha *et al.*, 2021 ▸). Thus, the electron scattering factors of charged atoms need careful treatment and parametrization (Yonekura & Maki-Yonekura, 2016 ▸). As a result, the obtained electrostatic potential maps may have negative or zero values at negatively charged functional groups; for example, it was observed that the amplitudes of the peaks corresponding to phosphate groups in RNA are significantly smaller than the peaks representing the bases (Wang & Moore, 2017 ▸).

A frequently used and the simplest model applied in X-ray diffraction, called the independent atom model (IAM), is based on spherical scatterers located at atom positions. More sophisticated and advanced methods involve aspherical modeling of the scatterer, such as the use of the multipole expansion in spherical harmonics. One of these methods, based on the Hansen–Coppens equation for modeling the electron density, served as the cornerstone of the data bank of aspherical atom types known as the University at Buffalo Data Bank (UBDB; Dominiak *et al.*, 2007 ▸; Jarzembska & Dominiak, 2012 ▸; Kumar *et al.*, 2019 ▸). Currently, a successor to the UBDB is being developed under the name the Multipolar Atom Types from Theory and Statistical clustering (MATTS) data bank (Jha *et al.*, 2022 ▸; Rybicka *et al.*, 2022 ▸). Apart from UBDB, two other data banks of electron-density parameters for atom types used in X-ray crystallography have gained significant popularity: ELMAM (Pichon-Pesme *et al.*, 2004 ▸; Domagała *et al.*, 2012 ▸) and Invariom (Dittrich *et al.*, 2013 ▸). The usage of such atom types to recreate the electron density of the sample in an accurate way is justified as its parameters, derived from theoretical or experimental atom positions in one chemical environment, can be used in a similar chemical environment: these are transferable aspherical atom model (TAAM) parameters. The superiority of TAAM over the simple IAM has been proven over the years (Bąk *et al.*, 2011 ▸; Dittrich *et al.*, 2006 ▸, 2013 ▸; Jha *et al.*, 2020 ▸). TAAM has also been used to provide a deeper understanding of the electrostatic inter­actions within many protein and nucleic acid systems (Malińska *et al.*, 2014 ▸; Kulik *et al.*, 2015 ▸; Kumar & Dominiak, 2021 ▸). Even though UBDB was originally designed for X-ray scattering, it can be applied to electron scattering by using the Mott–Bethe formula without introducing any modifications to the data bank. To date, refinement of small molecules with electron scattering factors has been performed by us for carbamazepine (Gruza *et al.*, 2020 ▸) and for glycine (Jha *et al.*, 2021 ▸). It was found that for an O atom in a carboxylate group the scattering function is negative at resolutions worse than ∼9 Å (see Fig. S1 in Jha *et al.*, 2021 ▸). At such low resolutions this O atom would generate a fully negative (in the entire volume around the atom) electrostatic potential.

This work is the first attempt to calculate the electrostatic potential maps of protein crystals with sophisticated models of electron scattering based on a multipole approach. We focus on two model proteins solved with 3D ED at a relatively high resolution of close to 1.8 Å. We calculate the electrostatic potential maps using TAAM, based on the UBDB atom types, and we compare these maps with those calculated with IAM and with the maps deposited in the RCSB PDB for the same structures. We draw conclusions on the effects of introducing partial charges (such as the charge at the O atom in a carboxylate group) and aspherical deformation of the electron density during the map-calculation process at a resolution of ∼1.8 Å. We also relate the features visible in those maps to the features that are visible in electron-density maps calculated at the same resolution, including thermal smearing effects.

## 2. Methods

### 2.1. Theoretical background

In order to derive the electron scattering factors in an analytical representation, two approaches may be applied. In the first approach, the electron scattering factors are calculated directly using quantum-chemistry methods and are presented in the form of tabulated numerical one-dimensional grids. Next, the set of Gaussian functions is fitted to obtain an analytical representation. The second approach uses the X-ray diffraction scattering factors in the form of tabulated numerical one-dimensional grids, which are approximated by a sum of Gaussian functions to arrive at the analytical representation and then finally converted to electron scattering factors with the Mott–Bethe formula. The first approach is considered to be more correct (Peng *et al.*, 1996 ▸). However, neither of these two approaches can be applied at any resolution, as detailed in Colliex *et al.* (2006 ▸). On the other hand, the multipole model based on the Hansen–Coppens formalism uses the sum of Slater functions and spherical harmonics to parametrize the electron density, which corresponds to using spherical Bessel functions and spherical harmonics to parametrize the X-ray scattering factors. Analytical expressions for the electron scattering factors can then be derived directly without approximations. This is the reason why we can use the same data bank of electron-density multipolar parameters to obtain X-ray and electron scattering factors in an analytical representation that is applicable at any resolution.

#### 2.1.1. Independent atom model (IAM)

The scattering potential and electrostatic potential produced by the electrons scattered by a sample are considered equivalent (Peng, 1999 ▸). High-energy elastic electron scattering from a group of well separated atoms generates the Coulomb electrostatic potential *V*(**r**), which depends not only on the distribution of the electron density ρ_*n*_(**r**′) but also on the positions of the atomic nuclei **R**_*n*_ and the atomic number *Z* (Ghermani *et al.*, 1993 ▸): 

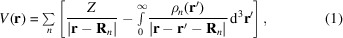




According to the kinematical approximation, we assume that the electron scattering amplitudes are proportional to the Fourier transform of the potential distribution (Cowley, Goodman *et al.*, 2006 ▸). To calculate the spherical electron scattering factors 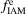 , it is possible to use the spherical X-ray scattering factors 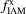 calculated with a quantum-mechanical method such as the atomic multiconfiguration Dirac–Fock code (Rez *et al.*, 1994 ▸) and then use the Mott–Bethe formula based on the Born approximation (Mott & Massey, 1965 ▸): 

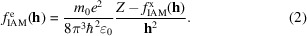




Here, |**h**| = 2sin(θ)/λ, where θ and λ represent half of the scattering angle and the electron wavelength, respectively. *m*_0_ and *e* are the mass and electron charge, whereas ɛ_0_ is the permittivity of vacuum. The electron scattering factors have been parametrized for all neutral atoms (Peng *et al.*, 1996 ▸) and are gathered in *International Tables for Crystallography* (Colliex *et al.*, 2006 ▸) as the *a*_*i*_ and *b*_*i*_ values of the approximations with the sums of five Gaussians.

#### 2.1.2. Transferable aspherical atom model (TAAM)

Firstly, let us look at the multipole model of electron density, based on the Hansen–Coppens equation, in which the total atom charge density is divided into three terms: spherical core-electron and valence-electron terms ρ_core_ and ρ_val_, represented by Slater-type functions, and an aspherical multipole expansion term, represented by both Slater-type radial functions (*R*_*l*_) and a finite spherical harmonic expansion in a nucleus-centered local frame (*Y*_*lm*_) (Hansen & Coppens, 1978 ▸): 

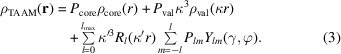




The ρ_core_ and ρ_val_ terms are spherically averaged, normalized to one electron, and have to be multiplied by *P*_core_ and *P*_val_ parameters representing the electron populations of core and valence electrons. *P*_*lm*_ represents the population of multipole densities. The κ and κ′ parameters reflect the expansion and contraction of the spherical valence shell and the aspherical part, respectively. The atomic multipolar scattering factors for X-ray scattering 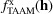 can be derived based directly on the parameters from the Hansen–Coppens equation (Hansen & Coppens, 1978 ▸): 

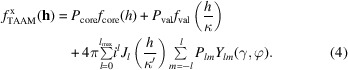




*f*_core_(*h*) and* f*_val_(*h*/κ) represent the atomic form factors from the core and spherically averaged valence-electron densities, whereas *J_l_* (*h*/κ′) denotes the* l*th-order Fourier–Bessel transforms of Slater radial functions. The Mott–Bethe formula (2) can then be used to transform the aspherical X-ray scattering factors 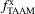 to the aspherical electron scattering factors 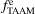 in a similar way as in the IAM but without using approximations with Gaussian fitting.

#### 2.1.3. Structure factors

For both IAM and TAAM, the structure factors *F*_e_(**h**) for a crystal in an electron diffraction experiment can be expressed using a standard formula that is also valid for X-ray or neutron diffraction with appropriate form factors (Rupp, 2009 ▸; Cowley, Goodman *et al.*, 2006 ▸; Chodkiewicz *et al.*, 2018 ▸). In this formula, the temperature factor for thermal vibrations depends on the Debye–Waller *B* factor. The *B* factor may represent atomic motion and possible static displacive disorder described by isotropic or anisotropic displacement parameters (Trueblood *et al.*, 1996 ▸). In the case of proteins, we usually focus on isotropic *B* factors.

#### 2.1.4. Apparent change in *B* factors

Whenever the scattering model is replaced with a new model, it is expected that the overall scale factor and overall *B* factor may change. This change can be quantified from the equation 

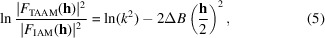

where *k* is a scale factor between |*F*_TAAM_(**h**)| and |*F*_IAM_(**h**)|, whereas Δ*B* = *B*_IAM_ − *B*_TAAM_. Indeed, in X-ray diffraction studies of small organic molecules and proteins, refinements using more accurate scattering models than IAM showed improved *B* factors and geometries (Brock *et al.*, 1991 ▸; Pichon-Pesme *et al.*, 1995 ▸; Jelsch *et al.*, 1998 ▸; Afonine *et al.*, 2004 ▸, 2007 ▸; Dittrich *et al.*, 2005 ▸; Dominiak *et al.*, 2007 ▸; Woińska *et al.*, 2016 ▸; Jha *et al.*, 2020 ▸). It was found that in X-ray diffraction refinement with IAM for a small-molecule crystal at 0.8 Å resolution, the anisotropic displacement parameters are overestimated by 25–35% with respect to the reference data. In the case of electron diffraction data at 0.8 Å resolution, the anisotropic displacement parameters of non-H atoms generated with IAM were about 40% smaller compared with the reference neutron data (Gruza *et al.*, 2020 ▸).

### 2.2. Calculations

Experimental 3D ED data sets containing electrostatic potential maps with fitted atomic coordinates for the lysozyme (*Gallus gallus*) structure at 1.8 Å resolution (PDB entry 5k7o, EMDB entry EMD-8217; de la Cruz *et al.*, 2017 ▸) and for proteinase K (*Parengyodontium album*) at 1.75 Å resolution (PDB entry 5i9s, EMDB entry EMD-8077; Hattne *et al.*, 2016 ▸) were downloaded from the RCSB PDB (Berman *et al.*, 2000 ▸) and the Unified Data Resource for 3DEM database (Lawson *et al.*, 2016 ▸). The maps for EMDB entries EMD-8217 and EMD-8077 were based on the experimental reflection data sets with 96.83% and 94.12% completeness, respectively. The voxel size of both maps was ∼0.6 Å. To build chemically valid models of the studied crystal structures, H atoms were added to the protein structures based on geometry with *MolProbity* (Williams *et al.*, 2018 ▸) and adjusted to ensure the catalytically competent protonation state at pH 4.7 for lysozyme and pH 8 for proteinase K. H atoms in water molecules were added with *UCSF Chimera* (Pettersen *et al.*, 2004 ▸), considering clashes and hydrogen-bond formation. The lengths of all covalent bonds engaging the H atoms were extended to match the typical distances observed in neutron diffraction data as they are considered to be more accurate (Nakane *et al.*, 2020 ▸). It is known that protons make an important contribution to the electrostatic potential. In proteinase K, the side chain of the partially missing residue Arg64 was rebuilt in *Maestro* 11.9 (Release 2019-1, Schrödinger). Atomic *B* factors equal to 120% of the *B* factors of the closest non-H atoms were assigned to all H atoms in proteins except for methyl-group H atoms. In the case of H atoms in methyl groups and water molecules, 150% of the *B* factors of the closest non-H atoms were used (Lübben *et al.*, 2014 ▸). Next, the file format was changed to *SHELX* style (Sheldrick, 2015 ▸) with *Mercury* (Macrae *et al.*, 2020 ▸) and *LSDB* (Volkov *et al.*, 2004 ▸) was used to transfer the UBDB2018 atom-type parameters (Kumar *et al.*, 2019 ▸). The presence of all H atoms and the Arg64 atoms was essential for the correct atom-type assignment. In the proteinase K structure, the UBDB parameters (multipole model parameters *P*_val_, *P*_*lm*_, κ and κ′) were manually adjusted for the S atoms in sulfate molecules and for water molecules 401, 408 and 480. These atom types were not recognized correctly via the automatic assignment as the atom types were missing or the distances between atoms were too small. The latter water molecule was located at the symmetry element, so the multipole parameters were multiplied accordingly to the site multiplicity, in this case by 1/2.

The reflection indices necessary for structure-factor calculations were generated with *Python* 3.7 with 100% completeness up to the given resolution and with additional reflections filling a cubic shape, taking the experimental unit-cell dimensions into account. An exemplary *Python* script to generate the reflection indices for lysozyme is provided in Section S1. The artificial reflection indices were truncated to the desired resolution and superfluous reflections in the ‘corners’ of the cube were removed in the software, which was an extension of the DiSCaMB library available at http://4xeden.uw.edu.pl/software/ (Chodkiewicz *et al.*, 2018 ▸). Using the same software, structure factors were calculated for X-ray diffraction for the xTAAM model with UBDB2018 parameters. These were then converted using the Mott–Bethe formula (2) to arrive at the eTAAM model for electron diffraction. The coefficients for analytical Gaussian approximation to scattering factors for the xIAM model for X-ray scattering (Doyle & Turner, 1968 ▸; Fox *et al.*, 1989 ▸) and eIAM for electron scattering (Peng *et al.*, 1996 ▸) were directly taken from Brown *et al.* (2006 ▸) and Cowley, Peng *et al.* (2006 ▸), respectively. The calculated maps (Fourier maps) for eTAAM, eIAM, xTAAM and xIAM were generated in the *XDFOUR* module of the *WinXD*2016 package (Volkov *et al.*, 2016 ▸) at resolutions of 1.8 Å for lysozyme and 1.75 Å for proteinase K and with a voxel size of 0.3 Å. The map format was changed to Situs format using in-house scripts for visualization in *UCSF Chimera* (Pettersen *et al.*, 2004 ▸). To arrive at e Å^−1^ units, the calculated potential maps values were recalculated (for more details, see Section S2). It was checked that decreasing the completeness to 96.83% for lysozyme and to 94.12% for proteinase K had only a minor effect on the calculated electro­static potential maps, as shown in Supplementary Fig. S1. Experimental structure-factor amplitudes were only used to generate the Wilson plots. Our aim was to generate the theoretical maps based only on the atom positions and *B* factors without the need to use any additional data, including the structure-factor or reflection indices derived from the experiment. The 2D *F*_TAAM_ − *F*_IAM_ deformation maps were calculated in the *XDFOUR* module of the *WinX*D2016 package (Volkov *et al.*, 2016 ▸) at 1.8 Å resolution using a 0.1 Å voxel size. The experimental density maps deposited in the Unified Data Resource for 3DEM were used for comparison of the electrostatic potential map features with the calculated maps.

### 2.3. Analysis

Two different approaches to data analysis were applied. The first approach was based on data in real space, taking into account the experimental deposited 3D ED density maps EMDB entries EMD-8217 (de la Cruz *et al.*, 2017 ▸) and EMD-8077 (Hattne *et al.*, 2016 ▸) and the voxel values of the calculated electrostatic potential and electron-density maps. For visualization, all of the density maps were cut around the protein with a 3 Å margin, scaled to match the standard deviation of the voxel values of the experimental density maps and the mean voxel value was shifted to zero. The standard deviations for the lysozyme and proteinase K maps were 0.162 and 0.166, respectively. Cutting, scaling and visualization of the σ contours of the maps were performed in *UCSF Chimera* (Pettersen *et al.*, 2004 ▸). The calculation of the map correlation coefficients around the mean (CC) and the rank CC for the quantile rank-scaled maps (CCr) between two grid functions were based on equations (4) and (17) in Urzhumtsev *et al.* (2014 ▸). The calculations were performed in *Phenix* version 1.14 (Liebschner *et al.*, 2019 ▸) with the Map Sigma Level Comparison tool. To compare the experimental and calculated density maps in a quantitative manner close to atom positions, the covalent radius averaging method was used. It is more accurate than simple sampling at atom positions, especially when the voxel size of the map is large, because it takes more data points around the atom into account. In the covalent radius averaging method, averaging over grids sampled within the volume up to the covalent radius distance from atom positions is performed. Sampling was performed in *UCSF Chimera* every 0.1 Å using the original deposited experimental maps and the calculated and scaled IAM and TAAM maps. Further details of the sampling, together with the covalent atom radii for different elements and the resulting numbers of grid points, are available in Section S4. The rebuilt residue Arg64 in proteinase K was not taken into account in this analysis.

The second approach focused on the reciprocal-space information, with detailed analysis of the relations between calculated structure factors. Wilson plots were plotted with reciprocal squared resolution (1/*d* ^2^) shells averaged for each 0.01 Å^−2^ bin. In crystallography, the reliability factor (*R* factor) usually measures the agreement between the amplitudes of the structure factors from a model and from the diffraction data. Here, it was used to compare two models (TAAM and IAM). For the same purpose, the Fourier shell correlation (FSC) was calculated over all structure factors in 0.1 Å^−1^ reciprocal resolution (1/*d*) bins according to the formulae given in Harauz & van Heel (1986 ▸) and Nicholls *et al.* (2018 ▸).

## 3. Results and discussion

**Figure 1:**
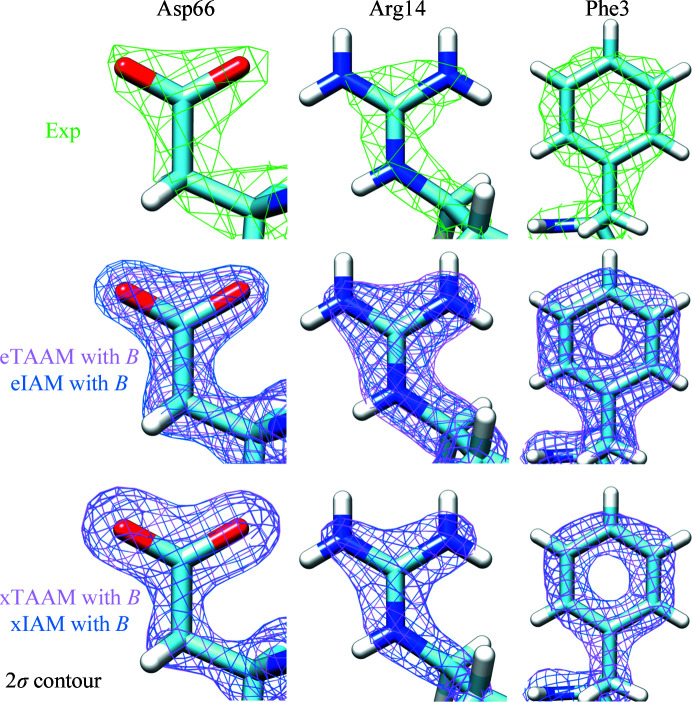
The contour electrostatic potential map for chosen amino-acid side chains from the lysozyme structure for experimental (Exp) and theoretical electrostatic potential maps (TAAM, based on the transferable aspherical atom model; IAM, based on the independent atom model) for the structure with PDB code 5k7o. eTAAM and eIAM maps were calculated using the electron scattering factors, whereas xTAAM and xIAM maps were calculated using the X-ray scattering factors. All of the maps are calculated at 1.8 Å resolution. The voxel values of all theoretical maps are scaled to the standard deviation of the experimental density map and the average value of zero, and their σ contours are then shown. The maps take thermal smearing effects into account (with *B*).

At a first glance, the 3D density maps shown in Fig. 1 ▸, calculated using the TAAM and IAM scattering factors, are very similar. The differences between the electrostatic potential maps based on the electron scattering factors (eTAAM and eIAM) are easily visible after taking a closer look at single amino-acid residues, in particular the charged residues such as Asp66 and Arg14. The electrostatic potential map contour encompasses a lower volume of positive electrostatic potential around O atoms in the negatively charged acidic side chains when the eTAAM is used in comparison with the eIAM. This is in agreement with our expectation that positive contributions to the Coulomb potential from the atomic cores (nuclei and core electrons) are partially canceled by the presence of excess valence electrons. This negative contribution from the O atom in the carboxylate group is much more subtle than one would expect from the scattering factors of oxygen in a carboxylate group. In fact, the oxygen scattering factor presented in Supplementary Fig. S1 of Jha *et al.* (2021 ▸) suggests that the contribution of this O atom to the electrostatic potential will be solely negative only at resolutions worse than 9 Å. For the positively charged Arg14 residue, the eTAAM map contour is larger around the N atoms than the eIAM contour. This means that the electrostatic potential from N atoms is more positive in eTAAM than is predicted by eIAM. The observations for Asp66 and Arg14 are typical for all negatively and positively charged amino acids in lysozyme and proteinase K. In contrast, the neutral Phe3 contours do not reveal visible differences between the eTAAM and eIAM. In the case of X-ray diffraction, the dependence of the electron-density shape on the atomic charges is negligible and the xTAAM and xIAM maps are strikingly similar at this countour level. This is also in line with theoretical expectations as X-ray scattering factors are always positive and are not influenced so much by charge differences.

**Figure 2:**
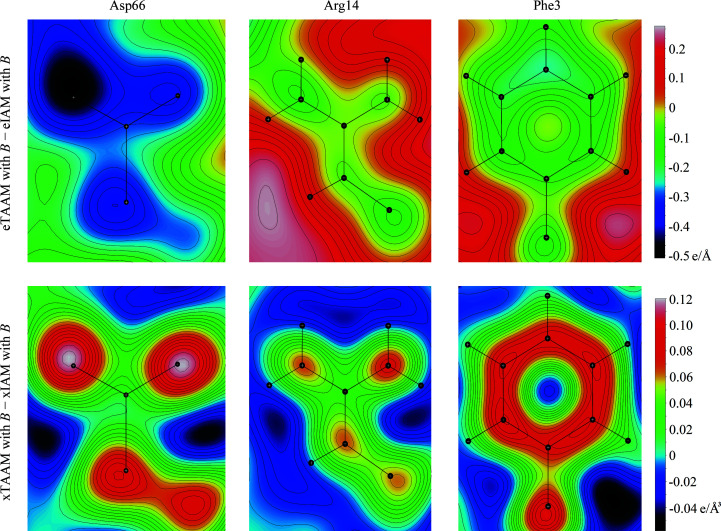
The 2D *F*_TAAM_ − *F*_IAM_ deformation density maps at 1.8 Å resolution for the same amino-acid side chains from the lysozyme structure PDB entry 5k7o as in Fig. 1 ▸. The maps take thermal smearing effects into account (with *B*). Note that values are given on the absolute scale.

Fig. 2 ▸ shows the 2D *F*_TAAM_ − *F*_IAM_ deformation density maps for the same residues as in Fig. 1 ▸ for electron and X-ray diffraction. The deviations between the two models are now clearly visible: the negative values for the carboxylate group indicate that the electrostatic potential values found around Asp66 in the eTAAM map are shifted towards more negative values than in the eIAM map. Note that the eIAM map only contains positive values. The most negative values of the deformation density map for eTAAM − eIAM, close to −0.51 e Å^−1^, are observed for the O atom of Asp66. The negative potential of the second O atom is compensated by the hydrogen bond to the hydroxyl group of Thr69. For Arg14, the deformation electrostatic potential values at N-atom positions are slightly negative, which means that the eIAM map contains more positive values than the eTAAM map. However, the deformation density at most of the H atoms on the surface of this group suggests more positive values in eTAAM than in eIAM. The left NH_2_ group is under the influence of the neighboring chloride anion. The deformation of the electrostatic potential map of Phe3 shows the presence of the same chloride anion at the top of the figure. On the other hand, the xTAAM − xIAM deformation electron-density maps do not show the presence of either the hydrogen bond in Asp66 or the adjacent chloride ion in Arg14 and Phe3. The deformation electron density is simply centered at the non-H-atom positions.

All of the maps in this work were calculated in two ways: with thermal smearing effects expressed by experimental *B*-factor values (with *B*) and without these effects (w/o *B*). The latter density maps, which are not physical, are presented in Supplementary Fig. S3. The general trends regarding the TAAM versus IAM comparison for the density maps without *B* factors are basically the same.

All of the maps shown in Fig. 1 ▸ are scaled to match the distribution of the values of the experimental 3D ED map and the average value of the voxel is equal to zero. It is then possible to qualitatively compare our theoretical maps with the electrostatic potential in the experimental maps, which were obtained using the experimental amplitudes and refined structural model. In general, all four theoretical density maps, from both electron and X-ray diffraction, are similar to the experimental map. The experimental map of the negatively charged carboxylic group more closely resembles the eTAAM and eIAM maps than the xTAAM and xIAM maps. Note that the experimental voxel size is close to 0.6 Å, whereas the voxel size of the theoretical maps is 0.3 Å. If we resample the calculated grids on 0.6 Å grids, it is possible to investigate the CC values around the mean between each pair of maps. The results for the experimental and theoretical lysozyme and proteinase K maps are shown in Supplementary Tables S2 and S3. CC values for both proteins between the full experimental and theoretical density maps range from 0.75 to 0.79. The CC values measured between the experimental and theoretical density maps extracted within the protein fragment with minimum solvent content are all higher than 0.92. Nevertheless, by looking at the CC it is not possible to differentiate the maps calculated with electron scattering factors from those calculated with X-ray scattering factors. Also, there is no significant difference in CC between the IAM and TAAM resampled maps or maps with or without *B* factors. To check whether the histogram equalization of the maps would provide new insights into the analysis, we have also added CCr to Supplementary Tables S2 and S3. The CCr values comparing the experimental and theoretical maps also do not reveal differences between the use of electron or X-ray scattering factors. Apparently, there is some factor that is not taken into account in any of the theoretical maps but that affects the experimental maps, which influences the map to a larger extent than the use of various scattering models or thermal smearing. This could be the lack of solvent modeling in the theoretical maps or unresolved solvent molecules inside the protein. Dynamic and inelastic scattering could also contribute to the discrepancy between theoretical maps and the maps based on experimentally determined intensities. A comparison of the CC and CCr values between different variants of the theoretical maps shows that all of the coefficients are much closer to 1.00 than in the case of correlations with the experimental maps. Nevertheless, in the case of electron diffraction we can see slightly higher differences between TAAM and IAM maps than in the case of X-ray diffraction.

**Figure 3:**
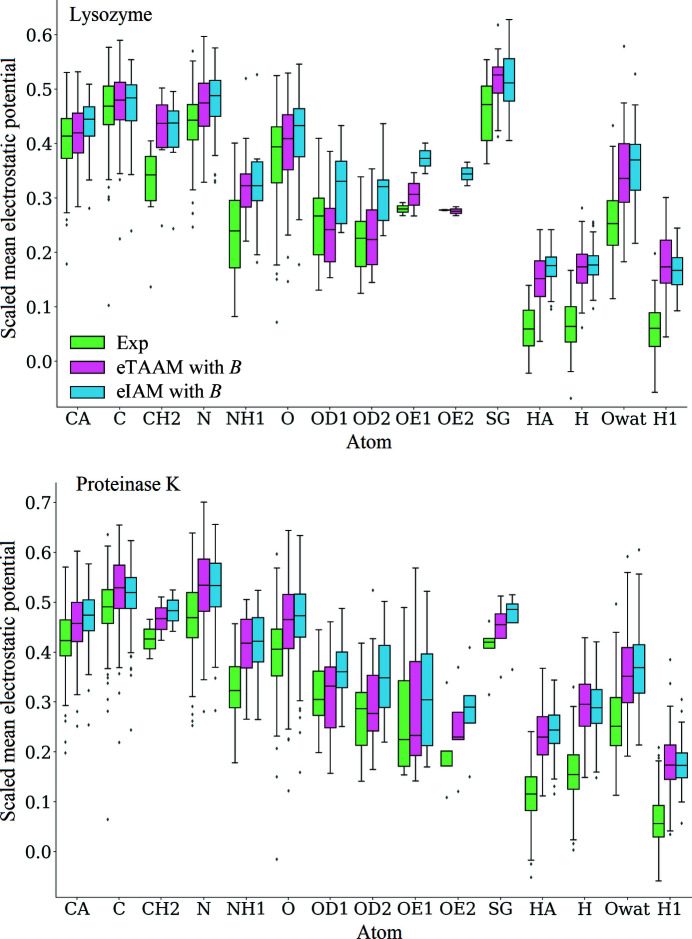
Boxplots for the average values of the electrostatic potential around chosen atom positions calculated for experimental (Exp) and scaled electrostatic potential maps (eTAAM and eIAM) for lysozyme and proteinase K. For details of the sampling and the choice of the atoms, see Section S4. All of the atom names follow the standard nomenclature present in the PDB structures of the proteins, except for the O atoms in the water molecules, which are here named Owat.

The correlation coefficients are not very sensitive to the changes in the density maps generated with TAAM/IAM, different *B*-factor treatment and electron/X-ray diffraction. To avoid the visual inspection of hundreds of amino acids in the various experimental and theoretical density maps, we performed a high-throughput analysis of the map values close to atom positions. The map values for atoms measured only at the atom positions are appropriate for analysis of maps with a small voxel size. When the voxel size is large, such as 0.6 Å, the values measured at atom positions would be prone to large variation. To avoid this effect, we sample the grid every 0.1 Å within the volume of a sphere with a radius specific for every element. For example, the values averaged for a CA atom would be calculated over 2103 grid points within 0.8 Å of the position of this CA atom. The details of the sampling method and the full list of covalent radii with the numbers of atoms in each structure are available in Section S4. Fig. 3 ▸ gathers the boxplots for the average density values in lysozyme and proteinase K for chosen atoms. For compatibility with the experimental density maps, the calculated eTAAM and eIAM maps were scaled. It can be seen that the eTAAM density maps tend to have closer values to the experimental maps than the eIAM maps, except for the H atoms, for which none of the models corresponded well to the experimental values. The differences between the eTAAM and eIAM are statistically significant, as shown by the Wilcoxon signed-rank test in Supplementary Table S4.

Such a high-throughput analysis of many amino acids allows us to follow certain trends in the unscaled density maps. Section S8 presents boxplots for the electrostatic potential (eTAAM and eIAM) and electron-density (xTAAM and xIAM) maps calculated for the same atoms in two systems, both with and without the thermal smearing effects, for lysozyme (Supplementary Fig. S4) and proteinase K (Supplementary Fig. S5). Not surprisingly, the diversity of the values of the maps taking into account the *B*-factor values in the calculations is higher than for the static models for non-H atoms. This is easily visible in the maps from electron and X-ray diffraction. Additionally, the mean eTAAM values are higher than the mean eIAM values, but for the maps derived with X-ray scattering factors this is not the case. The overall differences between the two models are small but consistent throughout the full data set. At the same time, the effect of switching between these two models in electron diffraction is larger than in X-ray diffraction. In contrast, in X-ray diffraction the effect of taking thermal smearing into account dominates over the change of the scattering model. The IAM does not take into account the deformation of the density arising from the influence of the local chemical environment. Note that averaging around the atom positions pictures a radial overview of the map features, whereas the TAAM is aspherical. Analysis of the density along the bonds would be more appropriate to obtain insight into the aspherical character of the density, but due to the large voxel size the sampling along the bonds would contain very few data points.

**Figure 4:**
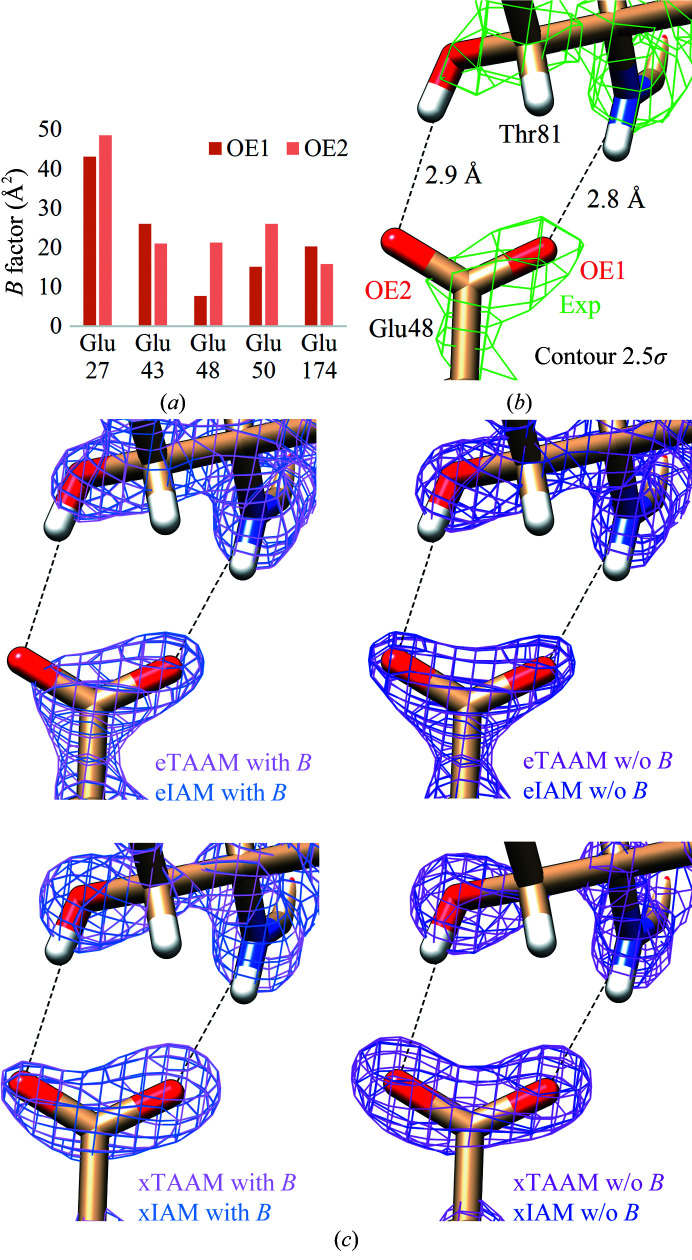
(*a*) *B*-factor values for the OE1 and OE2 atoms in Glu residues in proteinase K. (*b*) Experimental electrostatic potential density maps, (*c*) scaled theoretical electrostatic potential maps and (*d*) scaled theoretical electron-density maps for the Glu48 side chain with two hydrogen bonds marked with dashed lines. All density maps are shown at a 2.5σ contour.

Careful observation of the graphs in Supplementary Fig. S5 allows irregular behavior of the proteinase K density maps around OE1 atoms to be observed. The static maps are characterized by very small diversification of the eTAAM and eIAM map values, whereas the maps with thermal smearing show a large range of acquired values. A similar discrepancy, but to a much lower extent, is seen in the xTAAM and xIAM electron-density maps. Inspection of the *B*-factor values of the OE1 atoms in proteinase K reveals that there is one atom in Glu48 with a strikingly low *B* factor (Fig. 4 ▸). Visualizing the structural vicinity of this atom helps in understanding the differences that are visible in the previous graphs. This atom creates a hydrogen bond to the surrounding protein residues, is better stabilized and its movements are restricted. This more greatly influences the shape of the experimental and theor­etical scaled density maps in electron diffraction than in X-ray diffraction. The contours of the static density maps are not affected by the stabilizing influence of this hydrogen bond as they do not take the *B*-factor values into account in the calculations. This observation underlines the importance of having correctly determined *B* factors in structures deposited in the PDB. It is worth mentioning that no tools are currently used for *B*-factor validation in structures determined by cryo-EM and that such tools are urgently needed.

**Figure 5:**
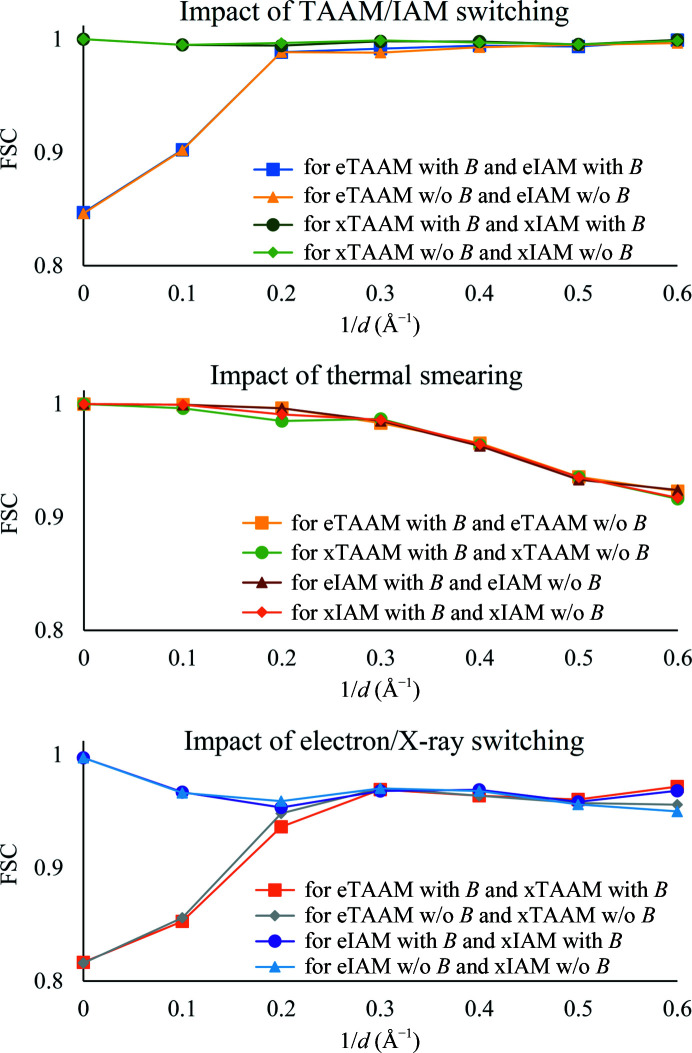
Fourier shell correlation calculated for structure factors in 0.1 Å^−1^ reciprocal resolution bins for various TAAM and IAM.

In order to quantify the impact of the TAAM/IAM, thermal smearing and the electron/X-ray scattering factors on the structure factors, we have calculated the *R* factors, which are presented in Supplementary Table S5 and analyzed in detail in Section S9. Analysis of the FSC, shown in Fig. 5 ▸, between different models allows us to follow the trends in the structure-factor values indicated by the *R* factor in separate resolution shells. Thus, on looking at the top panel of Fig. 5 ▸, it is straightforward that the highest deviations in the structure factors between eTAAM and eIAM with *B* factors are present in the low-resolution reflections. On the other hand, the introduction of thermal smearing affects the high-resolution structure-factor values. A very interesting trend in the structure factors is observed when electron or X-ray scattering factors are used. TAAM is more sensitive to the change from electron to X-ray scattering factors in the low-resolution region, while IAM is more sensitive in the medium- and high-resolution regions. This may suggest that the replacement of IAM by TAAM may influence the refinement procedure in a different way in electron diffraction data and X-ray diffraction data.

**Figure 6:**
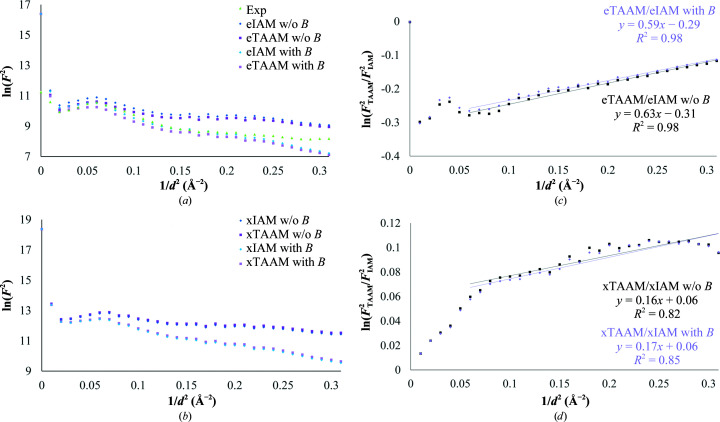
Wilson plots for lysozyme. (*a*) Electron and (*b*) X-ray diffraction structure factors. The experimental squared structure factors were scaled to match the local minimum value of the eTAAM with *B*. (*c*) Relation between squared eTAAM and eIAM structure factors with and without thermal smearing. (*d*) Relation between squared xTAAM and xIAM structure factors with and without thermal smearing. In all plots the resolution shells were averaged for each 0.01 Å^−2^ bin.

The Wilson plots show the squares of the structure factors generated for each model change with respect to the inverse square of the resolution (Figs. 6 ▸ *a* and 6 ▸ *b*). As expected, the higher the resolution, the lower the structure-factor amplitudes. A local minimum is observed in the region around 7 Å resolution, followed by a local maximum at around 4 Å resolution. The differences between the TAAM and IAM are most visible in the low-resolution region. The eTAAM squared structure factors are lower than the corresponding values for eIAM. However, the xTAAM and xIAM squared structure factors show the opposite trend and the differences are smaller. When including the effect of the *B* factors on the structure factors in the model calculation with thermal smearing the high-resolution structure factors diminish, and this is visible for both the calculated and the experimental structure factors.

It is possible to picture the relation between the squared eTAAM and eIAM structure factors (Fig. 6 ▸ *c*). The largest deviation appears in the region of resolution around 3.5–4.5 Å (∼0.08–0.05 Å^−2^) and appears again at low resolution. Including the thermal smearing effects in the calculations slightly impacts the slope of the fitted line. Then, by analysis of this slope and using (5), we can calculate the apparent change in *B* factors. For electron diffraction structure factors with thermal smearing Δ*B* = −1.18 Å^2^, while without thermal smearing Δ*B* = −1.24 Å^2^. The corresponding values for X-ray diffraction are −0.41 and −0.38 Å^2^, respectively (Fig. 6 ▸ *d*). These results show that the experimentally obtained *B* factors might be underestimated. However, this difference might be smaller than the standard uncertainties in the refined *B* factors of macromolecules.

Calculations of theoretical electrostatic density maps for macromolecules may potentially help in understanding the structural features of solved macromolecular complexes, such as the presence of charged ions and water molecules. Future work on this project includes calculations of electrostatic potential density maps at different resolutions with the comparison of chosen experimental data sets from 3D ED and cryo-EM.

## 4. Conclusions

We have developed a method to calculate theoretical electrostatic potential maps with high accuracy via structure-factor calculation. The method is based on a transferable aspherical atom model (TAAM) and is derived from the Hansen–Coppens multipole model with atom-type parameters transferred from the University at Buffalo Data Bank. The theor­etical TAAM maps for electron diffraction (eTAAM) at 1.8 Å resolution correspond well to the experimental density maps of lysozyme and proteinase K. The density maps based on the independent atom model (IAM), using the approximated electron scattering factors (eIAM), are not as sensitive to charged amino acids as the eTAAM maps. For comparison, we have also calculated the corresponding maps using X-ray scattering factors (xTAAM and xIAM, respectively). The density measured around atom positions reveal that in general the eTAAM maps show lower values than the eIAM maps, whereas the trend is opposite for the xTAAM and xIAM maps. Moreover, the differences between the eTAAM and eIAM maps are larger than those between the xTAAM and xIAM maps. The high-throughput analysis of densities measured around atoms in amino acids can reveal interesting structural features. The *B* factors may affect electrostatic potential maps in a different way than in the case of electron-density maps, for example in the presence of a hydrogen bond. 

## Supplementary Material

Supporting information Sections S1-S9, including Supplementary Figures and Tables. DOI: 10.1107/S2059798322005836/rr5218sup1.pdf
