# Supplementary material for: Theoretical 3D electron diffraction electrostatic potential maps of proteins modeled with a multipolar pseudoatom data bank
Source: Acta Crystallogr D Struct Biol. 2022 Jul 14;78(Pt 8):1010–20. doi: 10.1107/S2059798322005836 (PMC9344478; doi:10.1107/S2059798322005836)
Supplement: Supplementary file 1 [file d-78-01010-sup1.pdf]

STRUCTURAL BIOLOGY

Volume 78 (2022)

Supporting information for article:

Theoretical 3D electron diffraction electrostatic potential maps of proteins modeled with a multipolar pseudoatom data bank

Marta Kulik, Michał Leszek Chodkiewicz and Paulina Maria Dominiak

S1 Generation of reflection indices for lysozyme

In a typical diffraction experiment, the reflection intensities and their indices are collected. In order to calculate the theoretical structure factors for a given crystal, the indices of the reflections in the reciprocal space up to a given resolution are essential. Here, we calculate the reflection indices that correspond to the lysozyme crystal structure PDB ID 5k7o with the experimental unit cell parameters $a = 76.232, b = 76.232, c = 37.141, \alpha = \beta = \gamma = 90.00$ and space group P 4_3 2_1 2. The reported resolution for this structure was 1.8 Å. Since the indices depend also on the space group and are redundant, it is not necessary to calculate them for the full Ewald sphere. The reflection file is generated in sortav.hkl format and assigns the same intensity for all reflections (the intensity value is not used further by our programs). The systematic absences are not taken into account for simplicity.

```
# Python script to generate a file with reflection indices
# corresponding to PDB ID 5k7o
# Author: Marta Kulik

# resolution in Angstroms:
d=float(1.8)

# unit cell dimensions in Angstroms:
a=float(76.232)
b=float(76.232)
c=float(37.141)

import math

start_h = math.floor(-a/d)
end_h = math.ceil(a/d)
start_k = 0
end_k = math.ceil(b/d)
start_l = 0
end_l = math.ceil(c/d)
```

```
print("Reflection␣file␣will␣contain␣indices:")
print("h␣from␣",start_h,"␣to␣",end_h)
print("k␣from␣",start_k,"␣to␣",end_k)
print("l␣from␣",start_l,"␣to␣",end_l)

with open(r"sortav.hkl","w") as file1:
    for h in range(start_h,end_h+1):
        for k in range(start_k,end_k+1):
            for l in range(start_l,end_l+1):
                file1.write("%4s%4s%4s␣1100.00␣␣␣1.000␣␣␣1"%(h,k,l)+'\n')
    file1.write("␣␣␣0␣␣␣0␣␣␣0␣␣␣␣0.00␣␣␣0.000␣␣␣0\n")
```

S2 Recalculation of the electrostatic potential density

The electrostatic potential density maps are initially calculated by our programs in $Å^{-2}$, which is a result of old unit convensions. The calculated potential density maps values were scaled by a factor of 47.87801 to bring it to Volts as shown in the International Tables for Crystallography (2006), Vol. C, Chapter 4.3. Then recalculation from Volts to the e/Å units was done by a multiplying by a factor of 0.0694461541776244, taking into account the atomic unit of electric potential and length as in 2018 CODATA recommended values. Together, all the voxel values in the maps were multiplied by 3.32494366417784.

S3 Completeness

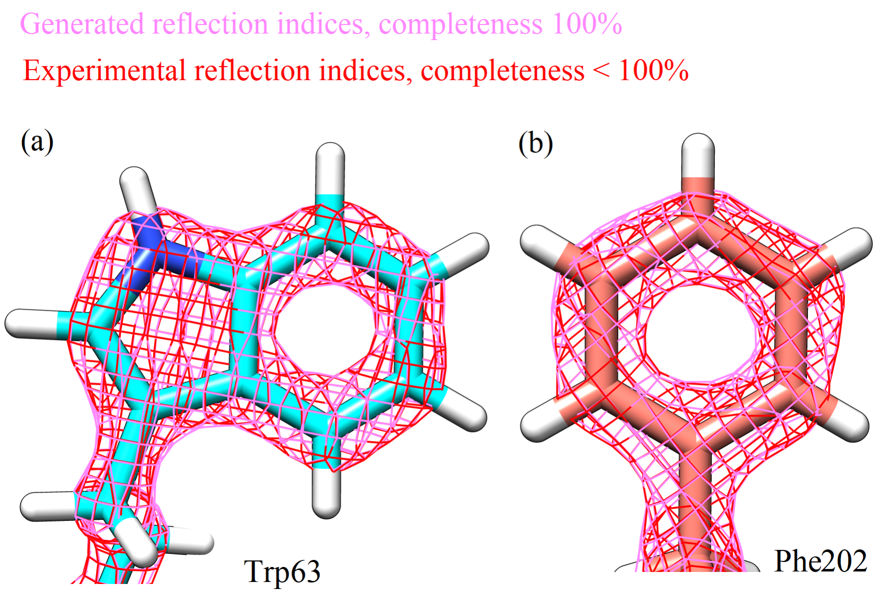

Fig. S1. Electrostatic potential density maps calculated using the electron scattering factors and based on the Transferable Aspherical Atom Model (eTAAM) of the (a) lysozyme structure at 1.8 Å, (b) proteinase K structure at 1.75 Å resolution.The experimental structures factor indices were deposited for both datasets with 96.83% and 94.12% completeness, respectively. The reflections indices from those datasets were taken to calculate the structure factors for the theoretical TAAM maps presented here in red. The maps shown in pink were generated based on the structure factors with reflection indices with 100% completeness. The maps are encompassing the region 15 Å from the atom CD2 and CG, respectively. The voxel values of all calculated maps are scaled to the standard deviation equal to 1 and the average value of 0, their 2 sigma contours are shown. The maps take into account the thermal smearing effects.

S4 The covalent radius averaging method

Here, we present a simple method to compare the experimental and theoretical density maps in a quantitative manner close to atom positions. This method is more accurate than sampling the density values at atom positions. The averaging of voxel values over the grids sampled within the covalent radius distance from atom positions is performed. Schematic justification for using the covalent radius averaging method is shown in Figure S2. Since the voxels in the experimental map are large, the assessment of the density map at the atom position only, marked with a black cross, depends mostly on the position of the voxels with respect to the atomic structure. By sampling the density map within the covalent radius distance for each atom (here 0.6 $\AA$ for oxygen), many sampled grid points make the assessment more accurate. The sampling radius is different for each element and it results in a different number of the sampled grid points, as shown in Table S1. The encompassed density is sampled every 0.1 $\AA$ within the covalent radius of each atom. Calculated density maps are calculated using 0.3 $\AA$ voxels.

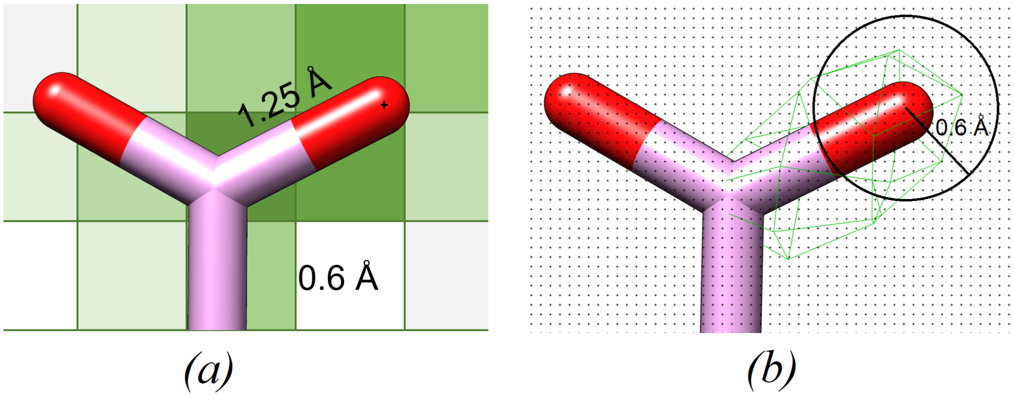

Fig. S2. A) Grid of the experimental map is ca. 0.6 $\AA$, which is close to half of the C-O bond in the glutamic acid side chain. B) Sampling done every 0.1 $\AA$, within the covalent radius (0.6 $\AA$) from the indicated oxygen atom. The experimental map 2 sigma contour is shown as green lines.

Table S1. *List of atoms for which the average values of the density map around atom positions were calculated. The atom names are the same as in the PDB structures of proteins except for the Owat atom that stands for the oxygen atom in water molecules.*

			Number of atoms	
Atom name	Radius [Å]	Number of grid points	Lysozyme	Proteinase K
CA	0.8	2103	129	278
C	0.8	2103	129	278
CH2	0.8	2103	6	2
N	0.7	1365	129	278
NH1	0.7	1365	11	11
O	0.6	895	129	278
OD1	0.6	895	21	31
OD2	0.6	895	7	14
OE1	0.6	895	5	12
OE2	0.6	895	2	5
SG	1	4139	8	5
HA	0.3	93	117	245
H	0.3	93	126	268
Owat	0.6	895	87	133
H1	0.3	93	87	133

S5 Density maps without B-factors

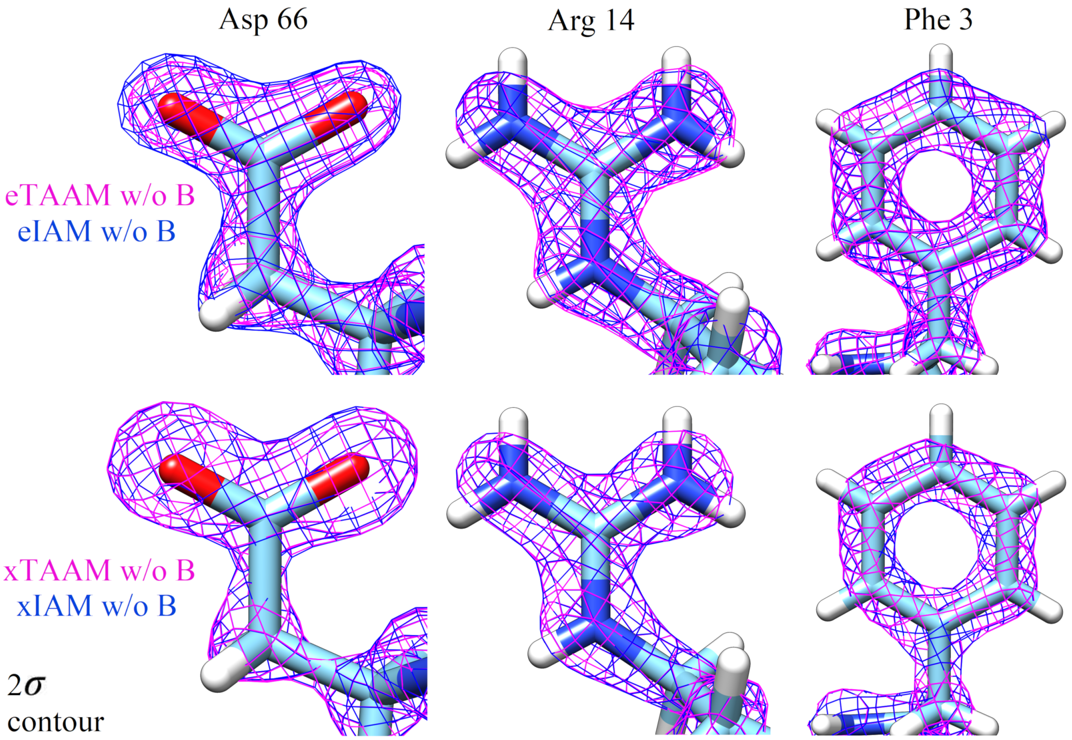

Fig. S3. Theoretical electrostatic potential density maps (TAAM - based on the Transferable Aspherical Atom Model, IAM - based on the Independent Atom Model) of the lysozyme structure at 1.8 Å. eTAAM and eIAM maps were calculated using the electron diffraction scattering factors, whereas xTAAM and xIAM maps were calculated using the X-ray diffraction scattering factors. The voxel values of all theoretical maps are scaled to the standard deviation of the experimental density map and the average value of zero, then their sigma contours are shown. The contour electrostatic potential density map for chosen amino acid side chains from the lysozyme structure. The maps neglect the thermal smearing effects (w/o B).

S6 Map and rank correlation coefficients

Map correlation coefficient about mean (CC) and rank correlation coefficient (CC_r) between two density maps of lysozyme and proteinase K are presented in Tables S2 and S3. The calculation is done in two ways: for a full density map (a cube containing one protein molecule with solvent) and a protein fragment (a 10 $\AA$ cube centered on the chosen atom buried inside the protein, almost no solvent). In the case of lysozyme, the 10 $\AA$ cube was centered on the CA atom of Ile 55 residue, whereas in the proteinase K, the Ala 231 residue was chosen. Exp stands for the experimental density map, eTAAM and eIAM are the calculated electrostatic potential density maps with or without B-factors, scaled to match the standard deviation and the average value of the experimental map.

Table S2. *Map correlation coefficient about mean (CC) and rank correlation coefficient (CC_r) between two density maps of lysozyme.*

		Full density map		Protein fragment	
Map 1	Map 2	CC	CC_r	CC	CC_r
Exp	eTAAM with B	0.77	0.44	0.95	0.88
Exp	eTAAM w/o B	0.77	0.53	0.93	0.86
Exp	eIAM with B	0.79	0.41	0.97	0.90
Exp	eIAM w/o B	0.79	0.55	0.94	0.88
Exp	xTAAM with B	0.79	0.47	0.95	0.91
Exp	xTAAM w/o B	0.77	0.56	0.93	0.88
Exp	xIAM with B	0.78	0.45	0.95	0.91
Exp	xIAM w/o B	0.77	0.56	0.93	0.88
eTAAM with B	eIAM with B	0.97	0.85	0.98	0.95
eTAAM w/o B	eIAM w/o B	0.98	0.91	0.99	0.97
eTAAM with B	eTAAM w/o B	0.95	0.84	0.98	0.97
eIAM with B	eIAM w/o B	0.95	0.79	0.98	0.97
xTAAM with B	xIAM with B	1.00	0.99	1.00	1.00
xTAAM w/o B	xIAM w/o B	1.00	1.00	1.00	1.00
xTAAM with B	xTAAM w/o B	0.95	0.80	0.98	0.97
xIAM with B	xIAM w/o B	0.95	0.80	0.98	0.97
eTAAM with B	xTAAM with B	0.95	0.82	0.95	0.93
eTAAM w/o B	xTAAM w/o B	0.96	0.89	0.96	0.96
eIAM with B	xIAM with B	0.98	0.98	0.97	0.99
eIAM w/o B	xIAM w/o B	0.98	0.97	0.98	0.98

Table S3. *Map correlation coefficient about mean (CC) and rank correlation coefficient (CC_r) between two density maps of proteinase K.*

		Full density map		Protein fragment	
Map 1	Map 2	CC	CC_r	CC	CC_r
Exp	eTAAM with B	0.75	0.34	0.96	0.84
Exp	eTAAM w/o B	0.76	0.45	0.94	0.81
Exp	eIAM with B	0.76	0.33	0.95	0.84
Exp	eIAM w/o B	0.77	0.47	0.94	0.82
Exp	xTAAM with B	0.77	0.39	0.94	0.84
Exp	xTAAM w/o B	0.75	0.50	0.92	0.81
Exp	xIAM with B	0.76	0.36	0.94	0.84
Exp	xIAM w/o B	0.75	0.48	0.92	0.81
eTAAM with B	eIAM with B	0.98	0.86	1.00	0.99
eTAAM w/o B	eIAM w/o B	0.99	0.92	1.00	0.99
eTAAM with B	eTAAM w/o B	0.96	0.85	0.99	0.97
eIAM with B	eIAM w/o B	0.96	0.82	0.99	0.97
xTAAM with B	xIAM with B	1.00	0.99	1.00	1.00
xTAAM w/o B	xIAM w/o B	1.00	0.99	1.00	1.00
xTAAM with B	xTAAM w/o B	0.96	0.80	0.99	0.96
xIAM with B	xIAM w/o B	0.96	0.82	0.99	0.96
eTAAM with B	xTAAM with B	0.96	0.82	0.97	0.97
eTAAM w/o B	xTAAM w/o B	0.96	0.88	0.97	0.97
eIAM with B	xIAM with B	0.98	0.98	0.98	0.98
eIAM w/o B	xIAM w/o B	0.98	0.97	0.97	0.98

S7 Wilcoxon signed-rank test

Table S4. *The Wilcoxon signed-rank test for the electrostatic potential maps for the lysozyme and proteinase calculated with the Transferable Aspherical Atom Model (eTAAM), Independent Atom Model (eIAM) and experimental maps EMD-8217 and EMD-8077. The values taken for the analysis are identical to the values used to generate the boxplot graphs in Figure 3.*

		eIAM-eTAAM	eIAM-Exp.	eTAAM-Exp.
Lysozyme	Statistic	1483	372.5	2333
	P-value	8.80E-10	2.71E-19	1.24E-05
Proteinase	Statistic	9303	1350.5	5259
	P-value	3.80E-13	3.25E-41	6.09E-26

S8 Boxplots for TAAM vs IAM comparison

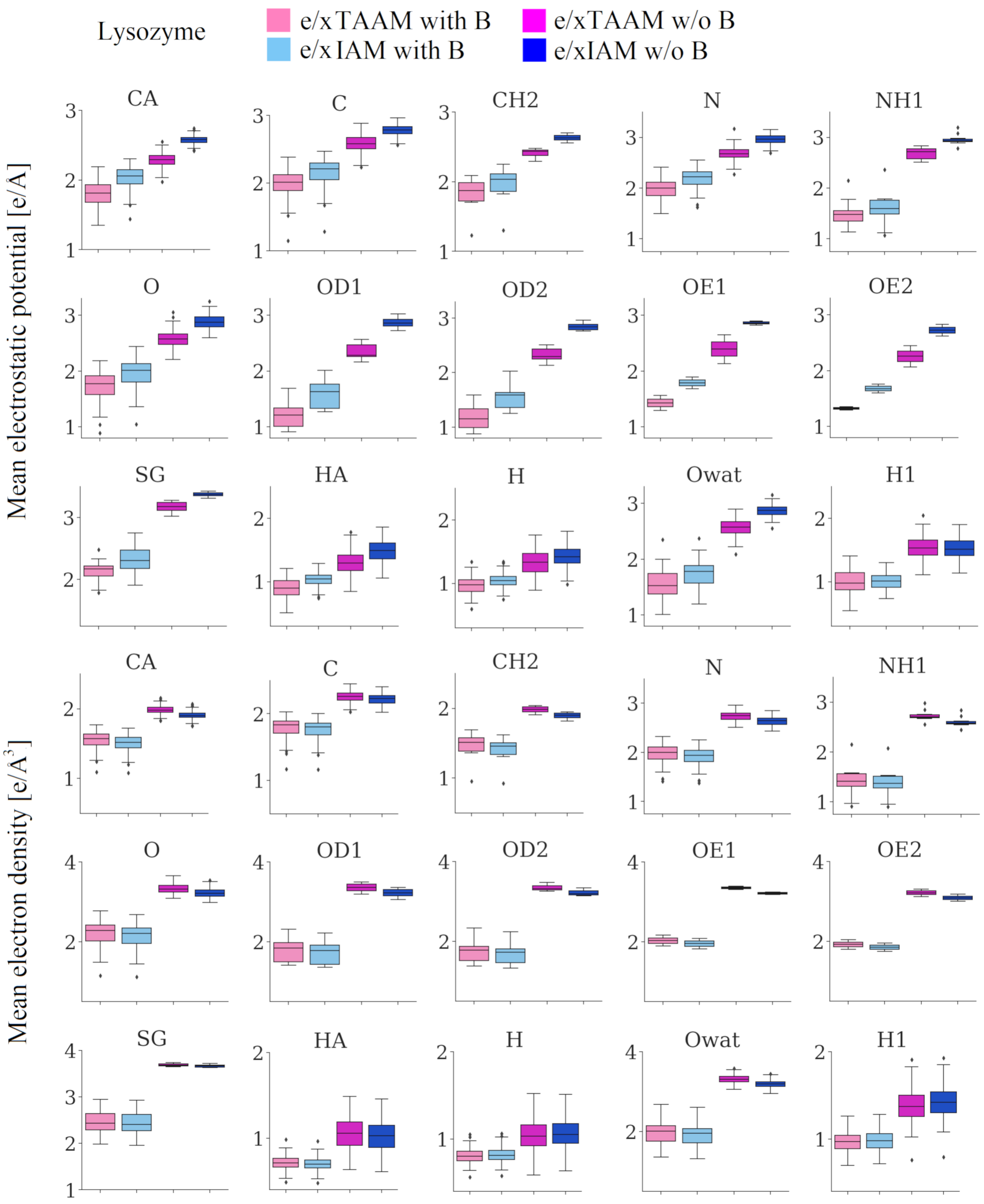

Fig. S4. Boxplots for the average values of the unscaled electrostatic potential (eTAAM and eIAM) and unscaled electron density (xTAAM and xIAM) around chosen atom positions in lysozyme. The light and dark colors indicate taking into account and neglecting the thermal smearing effects, respectively. All the atom names follow the standard nomenclature present in the PDB structures of proteins, except for the oxygen atoms in the water molecules, named here Owat.

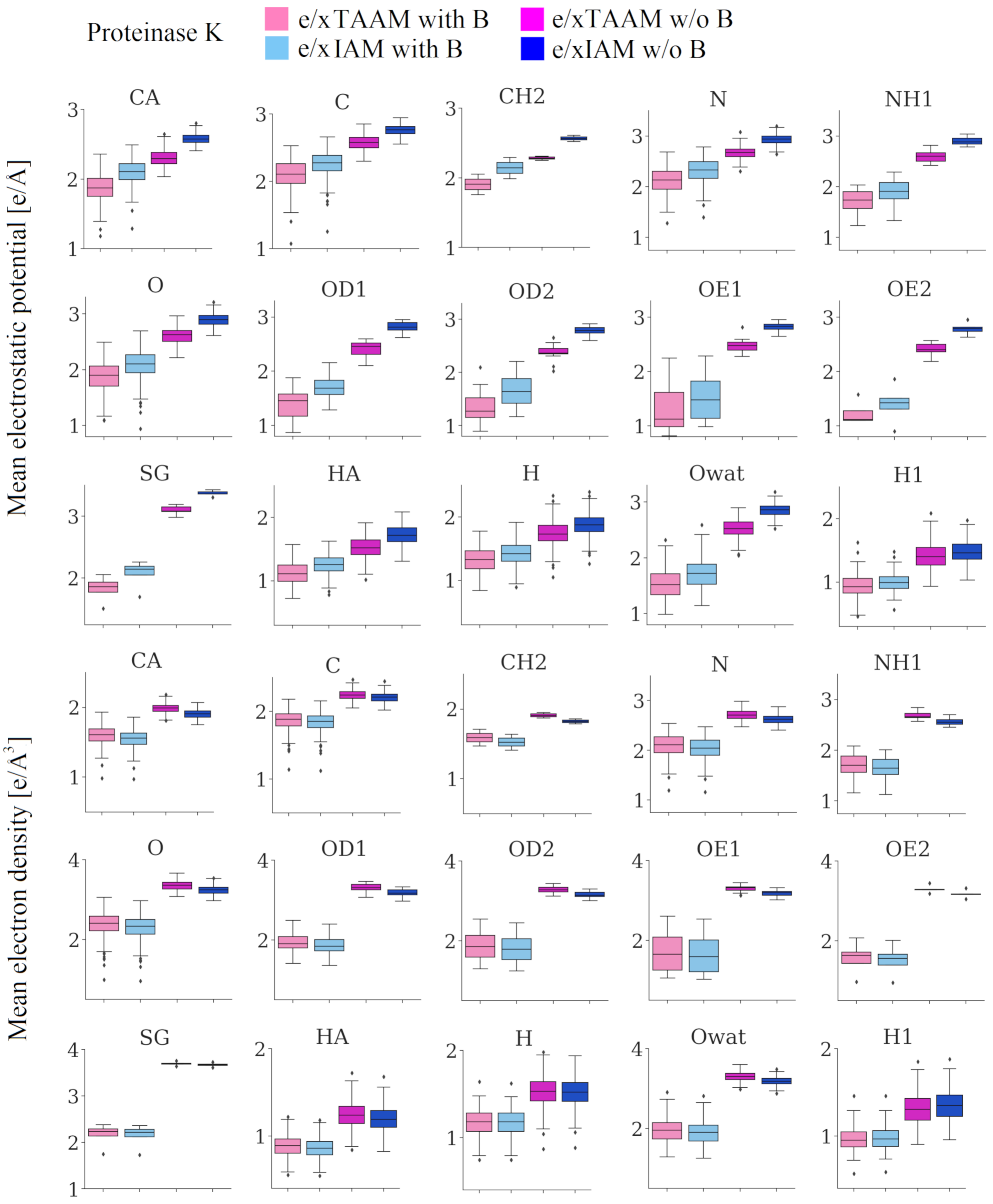

Fig. S5. Boxplots for the average values of the unscaled electrostatic potential (eTAAM and eIAM) and unscaled electron density (xTAAM and xIAM) around chosen atom positions in proteinase K. The light and dark colors indicate taking into account and neglecting the thermal smearing effects, respectively. All the atom names follow the standard nomenclature present in the PDB structures of proteins, except for the oxygen atoms in the water molecules, named here Owat.

S9 R factor analysis

In order to quantify the impact of the TAAM/IAM, thermal smearing and the electron/X-ray scattering factors on the structure factors, we have calculated the R factors, presented in Table S5. The point of this analysis was to order the impact of the latter variants of the calculations. The difference between eTAAM with B and eIAM with B, which is equal to 13%, can be contrasted with the value of R factor (Observed) reported by the authors of the original eIAM refinement: 24.16%. Note that all the R factor values mentioned in Table S5 come from calculations between pairs of models being at absolute scale, not from the refinement procedure. The R factors calculated between the eTAAM and eIAM structure factors are higher than those for xTAAM and xIAM, which underlines the fact that for electron diffraction the choice of the model plays a more significant role than for X-ray diffraction. Nevertheless, all the values are lower than 13% so the choice of the model does not apply huge changes to the structure factors. Upon applying thermal smearing, the structure factors show larger deviation, while the largest impact on the structure factors comes from switching between electron and X-ray scattering factors.

Table S5. *R factors (R_1 and R_2) calculated between different variants of the lysozyme structure factors.*

	F_1	F_2	$R_1 = \frac{\sum \lvert\lvert F_1\rvert - \lvert F_2\rvert\rvert}{\sum \lvert F_1\rvert}$	$R_2 = \frac{\sum \lvert\lvert F_2\rvert - \lvert F_1\rvert\rvert}{\sum \lvert F_2\rvert}$
Impact of the scattering model	eTAAM with B	eIAM with B	0.13	0.11
	eTAAM w/o B	eIAM w/o B	0.12	0.11
	xTAAM with B	xIAM with B	0.04	0.04
	xTAAM w/o B	xIAM w/o B	0.04	0.05
Impact of thermal smearing	eTAAM with B	eTAAM w/o B	0.61	0.38
	xTAAM with B	xTAAM w/o B	0.65	0.39
	eIAM with B	eIAM w/o B	0.61	0.38
	xIAM with B	xIAM w/o B	0.64	0.39
Impact of electron/ X-ray diffraction	eTAAM with B	xTAAM with B	2.39	0.71
	eTAAM w/o B	xTAAM w/o B	2.46	0.71
	eIAM with B	xIAM with B	1.95	0.66
	eIAM w/o B	xIAM w/o B	2.00	0.67
